# Undergraduate clinical posting in Psychiatry: Are we paying enough attention?

**DOI:** 10.4103/0019-5545.64585

**Published:** 2010

**Authors:** Shivanand Kattimani

**Affiliations:** Department of Psychiatry, Jawaharlal Institute of Postgraduate Medical Education and Research, Gorimedu, Pondicherry-605006, India. E-mail: drshivanand@gmail.com

Sir,

According to the recent guidelines set by Medical Council of India (MCI), it is now compulsory to have psychiatry posting for 14 days as part of rotatory internship, besides completing three hours per day for 14 days of psychiatry posting during the fifth or sixth semester for MBBS undergraduates (UGs). In many medical colleges, where there are postgraduate (PG) degrees in psychiatry, UG teaching is not given importance. The previous symposium on UG teaching in psychiatry has highlighted the importance of this issue very clearly.[1,2] There are 300 medical colleges in India that teach MBBS students; of these, 104 have PG courses in psychiatry.[3] Apart from having short term training programs for general duty medical officers (GDMO), to manage common psychiatric and substance use disorders in their practice, it is wise to show the same kind of interest and investment in training our UGs.

The medical colleges which do not have psychiatric departments can have their UGs posted to the nearest government psychiatric treatment centers. Research shows greater exposure to and working with mentally ill persons during medical training decreases fear and creates a positive attitude towards caring for the mentally ill.[4,5] The MCI has stated the goals and objectives of psychiatry training with a holistic approach.[6] Other additional goals which need to be developed are - the ability to make differential diagnosis, identify common side effects and information giving skills, either verbal or written, which include talking to patients or relatives or other specialty professionals about matters related to psychiatry. Their awareness of common psychotropics used and their indications, along with their dose and duration, can be enhanced to help them develop the ability to identify common side effects and manage them. We can enhance their knowledge on modified electroconvulsive therapy (MECT) procedure by demonstrating to small batches and informing them of its indications, myths, and adverse effects. We can teach principles of management of psychiatric emergencies-suicide threat and violence, one or two days in the out patient department (OPD) to see how follow-up is done. They can be introduced to use of classificatory systems in diagnosis, use of common rating or diagnostic scales and psychological tests. There has to be a separate slot for assessment and feedback or to discuss topics of their choice. There is a need to stress on physical examination in persons diagnosed with psychiatric disorders and detecting signs and symptoms of organicity in a person with psychiatric symptoms .They can be involved during their internship psychiatry posting working on common mental disorders as work up and discussing with consultants or taking up small presentations of psychiatric topics which are relevant for their entrance and general applicability, even when they work as GDMO or take specialty courses.

This effort helps in creating awareness about various mental disorders and in decreasing stigma attached towards psychiatry. We, as psychiatry professionals, need to devote time and , keep ourselves well versed with current knowledge about our subject and conduct ourselves in dignified way.
